# Mitochondrial Fission Factor (MFF) Inhibits Mitochondrial Metabolism and Reduces Breast Cancer Stem Cell (CSC) Activity

**DOI:** 10.3389/fonc.2020.01776

**Published:** 2020-10-22

**Authors:** Rosa Sánchez-Alvarez, Ernestina Marianna De Francesco, Marco Fiorillo, Federica Sotgia, Michael P. Lisanti

**Affiliations:** ^1^Division of Cancer Sciences, Faculty of Biology, Medicine and Health, School of Medical Sciences, University of Manchester, Manchester, United Kingdom; ^2^Translational Medicine, School of Science, Engineering and Environment (SEE), Biomedical Research Centre (BRC), University of Salford, Greater Manchester, United Kingdom; ^3^Department of Clinical and Experimental Medicine, University of Catania, and ARNAS Garibaldi, Catania, Italy

**Keywords:** mitochondrial fission factor, CSCs, mitochondrial metabolism, breast cancer, mitochondrial dynamics, oxidative metabolism, metabo-stemness, mitochondrial mass

## Abstract

Elevated mitochondrial biogenesis and metabolism represent key features of breast cancer stem cells (CSCs), whose propagation is conducive to disease onset and progression. Therefore, interfering with mitochondria biology and function may be regarded as a useful approach to eradicate CSCs. Here, we used the breast cancer cell line MCF7 as a model system to interrogate how mitochondrial fission contributes to the development of mitochondrial dysfunction toward the inhibition of metabolic flux and stemness. We generated an isogenic MCF7 cell line transduced with Mitochondrial Fission Factor (MCF7-MFF), which is primarily involved in mitochondrial fission. We evaluated the biochemical, molecular and functional properties of MCF7-MFF cells, as compared to control MCF7 cells transduced with the empty vector (MCF7-Control). We observed that MFF over-expression reduces both mitochondrial mass and activity, as evaluated using the mitochondrial probes MitroTracker Red and MitoTracker Orange, respectively. The analysis of metabolic flux using the Seahorse XFe96 revealed the inhibition of OXPHOS and glycolysis in MCF7-MFF cells, suggesting that increased mitochondrial fission may impair the biochemical properties of these organelles. Notably, CSCs activity, assessed by 3D-tumorsphere assays, was reduced in MCF7-MFF cells. A similar trend was observed for the activity of ALDH, a well-established marker of stemness. We conclude that enhanced mitochondrial fission may compromise CSCs propagation, through the impairment of mitochondrial function, possibly leading to a quiescent cell phenotype. Unbiased proteomic analysis revealed that proteins involved in mitochondrial dysfunction, oxidative stress-response, fatty acid metabolism and hypoxia signaling are among the most highly up-regulated in MCF7-MFF cells. Of note, integrated analysis of top regulatory networks obtained from unbiased proteomics in MCF7-MFF cells predicts that this cell phenotype activates signaling systems and effectors involved in the inhibition of cell survival and adhesion, together with the activation of specific breast cancer cell death programs. Overall, our study shows that unbalanced and abnormal activation of mitochondrial fission may drive the impairment of mitochondrial metabolic function, leading to inhibition of CSC propagation, and the activation of quiescence programs. Exploiting the potential of mitochondria to control pivotal events in tumor biology may, therefore, represent a useful tool to prevent disease progression.

## Introduction

Mitochondrial function is essential for supplying energy to fuel cancer cell growth and metastatic dissemination (1). Furthermore, enhanced mitochondrial metabolism has emerged as one of the novel features of cancer stem cells (CSCs), which exhibit tumorigenic and self-renewal properties, carry metastatic potential and provide resistance to anti-cancer therapies (2–4). In this regard, cancer cells biologically recapitulate certain stemness features, including anchorage-independent growth and higher tumor-initiating capacity, as well as increased mitochondrial mass, biogenesis and protein translation, across multiple tumor types (5). These observations, which clearly suggest a key role for mitochondria in CSC maintenance and propagation, are corroborated by the evidence that mitochondrial metabolic function and OXPHOS are augmented in cancer cells with stemness features, as compared to the non-stem cancer cell sub-population (6, 7). As a consequence of these findings, the ability of several classes of mitochondria-interfering agents to inhibit CSC dissemination has been explored, as reviewed in De Francesco et al. (8). In this regard, several antibiotics that impair mitochondrial protein translation, as a side effect, have been shown to be effective in depleting the CSC population *in vitro*, as well as in clinical studies (9–13). As such, a deeper understanding of mitochondrial biology in cancer would pave the way toward the identification of targeted therapeutics, aimed at selectively halting and eradicating CSCs.

Adequate mitochondrial function also depends on the intrinsic mechanical changes in the dynamic structure and architecture of these organelles (14, 15). For instance, changes in mitochondrial size and shape, as well as localization, are critical for the homeostasis of the energetic machinery. The main macro-mechanical events regulating mitochondrial architecture are represented by cycles of mitochondrial fusion and fission (fragmentation), that together constitute a mitochondrial network (16, 17). Given their important role in maintaining mitochondria homeostasis, fusion and fission are tightly regulated processes, whose de-regulation is associated with metabolic dysfunction, that may precede the establishment of pathological phenotypes (14, 17). In cancer, the elevated nutrient demands of proliferating cells are mainly fulfilled by drastic changes in energetic metabolism, largely supported by mitochondria, whose changes in function and shape are pivotal to tumor growth (1). Indeed, several oncogenic signals have been shown to influence the number of mitochondria, which is dependent on fusion-fission dynamics (15, 16, 18). Likewise, aberrant mitochondrial fission results in massive mitochondrial fragmentation, resulting in inhibition of oxidative metabolism and depletion of ATP (19). Mitochondrial Fission Factor (MFF) is an integral membrane protein of the outer mitochondrial membrane that serves as the main molecular mediator regulating mitochondria fragmentation (20). During fission, the cytosolic protein Drp1 is recruited to the mitochondrial surface via MFF and the proteins Fis1 (mitochondrial fission protein 1), MiD49 and MiD51 (mitochondrial dynamics proteins of 49 and 51 kDa, respectively) (21–23). Oligomeric Drp1 complexes are, thereafter, assembled into specific structures named *puncta*, which wrap around mitochondrial tubules, forcing them toward the fission reaction (24). Several studies have demonstrated that increased MFF expression is required for successive mitochondrial fission and is associated with disease states (25). Nevertheless, the role of MFF in regulating mitochondrial dynamics in breast cancer has not been thoroughly elucidated.

Herein, we provide evidence that aberrant MFF expression in breast cancer cells drastically inhibits mitochondrial mass and function, associated with a reduction of mitochondrial oxidative metabolism and ATP depletion. Proteomic profiling of MFF-overexpressing breast cancer cells reveals a phenotype associated with the inhibition of mitochondrial metabolism and a quiescent cell state. Biologically, MFF-overexpressing breast cancer cells exhibit impaired 3D mammosphere formation capacity, suggesting that the manipulation of mitochondrial structure and dynamics may represent a useful tool to control stemness traits in breast cancer.

## Materials and Methods

### Cell Culture

MCF7 human breast cancer cells were purchased from ATCC. Cells were cultured in Dulbecco's modified Eagle's medium (DMEM), supplemented with 10% heat-inactivated fetal bovine serum, 100 units/mL of penicillin, 100 μg/mL of streptomycin and 2 mM Glutamax (ThermoScientific), in a 37°C humidified atmosphere containing 5% CO_2_, unless otherwise noted.

### Lentiviral Gene Transduction

Lentiviral plasmids, packaging cells and reagents were from Genecopoeia. Forty-eight hours after seeding, 293Ta packaging cells were transfected with lentiviral vectors encoding the mitochondrial fission factor (MFF, EX-Z4766-Lv-105), or empty vector (EV, EX-NEG-Lv105), using the Lenti-PacTM HIV Expression Packaging Kit, according to the manufacturer's instructions. Two days post-transfection, lentivirus-containing culture medium was passed through a 0.45 μm filter and added to the target cells (MCF7 cells) in the presence of 5 μg/ml polybrene. Transduced MCF7 cells were selected with 2.5 μg/ml puromycin.

### Western Blotting

Western blotting of stably transduced MCF7 cells was used to evaluate the efficiency of transduction. Briefly, 70–80% confluent stably-transduced MCF7 cells (harboring MFF and the respective Ex-Negative control) were harvested in lysis buffer (10 mM Tris pH 7.5, 150 mM NaCl, 1% Triton X-100, and 60 mM n-octyl-glucoside), containing protease (Roche) and phosphatase inhibitors (Sigma) and kept at 4°C for 40 min with rotation. Lysates were cleared by centrifugation for 10 min at 10, 000 × g and supernatants were collected. Equal amounts of protein lysate, as determined by using the BCA protein assay kit (Pierce), were diluted in SDS sample buffer and dry-boiled for 5 min, prior to separation by SDS-PAGE using 4–20% acrylamide gels (Biorad). Samples were then blotted onto nitrocellulose membranes (Biorad), blocked in 5% milk in TBS-Tween 20 (P9416, Sigma) for 1 h and probed with antibodies directed against MFF (Abcam), or β-actin (Sigma), which was used as loading control. Bound antibodies were detected using a horseradish peroxidase-conjugated secondary antibody (ab6789 and ab6721, Abcam) and the signal was visualized using Supersignal West Pico chemiluminescent substrate (ThermoScientific).

### Mitochondrial Staining

Mitochondrial activity was assessed using the fluorescent probe MitoTracker Orange (CM-H2TMRos-reduced form) (ThermoFisher), whose accumulation in mitochondria is dependent upon membrane potential. Mitochondrial mass was determined using the fluorescent probe MitoTracker Deep-Red (ThermoFisher), which localizes to mitochondria regardless of mitochondrial membrane potential. Stably transduced MCF7 cells (harboring MFF or the Ex-Negative Control) were seeded for 48 h. After 48 h, the cells were incubated with pre-warmed MitoTracker staining solution (diluted in DMEM without serum to a final concentration of 10 nM) for 30 min at 37°C. All subsequent steps were performed in the dark. Cells were washed in PBS, harvested, and re-suspended in 300 μL of PBS/Ca^2+^Mg^2+^. Cells were then analyzed by flow cytometry using Fortessa (BD Bioscience). Data analysis was performed using FlowJo software. Results are the average of the mean of three independent experiments, were normalized to the control (Ex-Neg) and are expressed as percentages of mean fluorescence intensity. At least 4 biological replicates were performed in each experiment.

### Seahorse XFe96 Metabolic Flux Analysis

Real time oxygen consumption rates (OCRs) and Extracellular acidification rates (ECARs) were determined for stably transduced MCF7 cells using the Seahorse Extracellular Flux (XFe96) analyzer (Seahorse Bioscience, MA, USA). Briefly, 10,000 cells per well were seeded into XFe96 well cell culture plates and incubated overnight with complete medium to allow attachment. After 24 h of incubation, cells were washed in either pre-warmed XF assay media containing 2 mM glutamine, pH 7.4 for ECAR measurements or in XF assay media supplemented with 10 mM glucose, 1 mM Pyruvate, 2 mM L-glutamine and adjusted at 7.4 pH for OCR measurements. Cells were then maintained in 175 μL/well of XF assay media at 37°C, in a non-CO_2_ incubator for 1 h. During the incubation time, 25 μL of 80 mM glucose, 9 μM oligomycin, and 1M 2-deoxyglucose were loaded for ECAR measurement or 10 μM oligomycin, 9 μM FCCP, 10 μM rotenone, 10 μM antimycin A were loaded for OCR measurements, in XF assay media into the injection ports in the XFe96 sensor cartridge. Measurements were normalized by protein content, determined by SRB. Data sets were analyzed by employing XFe96 software and GraphPad Prism software, using Student's *t*-test calculations. Results are the average of the mean of three independent experiments normalized to the control and are expressed as percentages of mpH/min/SRB for ECAR or pmol/min/SRB for OCR measurements. At least six biological replicates were performed in each experiment.

### 3D Mammosphere Culture

A single cell suspension of stably transduced MCF7 cells was prepared using enzymatic (1x Trypsin-EDTA, Sigma Aldrich), and manual disaggregation (25 gauge needle) as previously described (26). Cells were plated at a density of 500 cells/cm^2^ in DMEM-F12 phenol free supplemented with B27, 20,ng/ml EGF and 1% Pen/Strep in non-adherent conditions, in culture dishes coated with 2-hydroxyethylmethacrylate (poly-HEMA, Sigma-Aldrich). Cells were grown for 5 days and maintained in a humidified incubator at 37°C. After 5 days in culture, spheres > 50 μm were counted using an eyepiece graticule. Results are the average of the mean of three independent experiments normalized to the control (Ex-Neg) and are expressed as percentage of cells plated which formed spheres. Three biological replicates were performed in each experiment.

### Aldefluor Assay

ALDH activity was assessed in stably transduced MCF7 cells, which were seeded in monolayer for 48 h. The ALDEFLUOR kit (StemCell Technologies) was used to isolate the population with high ALDH enzymatic activity by flow cytometry (Fortessa, BD Bioscience). Briefly, cells were harvested and incubated in 1 ml of ALDEFLUOR assay buffer containing ALDH substrate (5 μl/ml) for 40 min at 37°C. In each experiment, a sample of cells was stained under identical conditions with 30 mM of diethylaminobenzaldehyde (DEAB), a specific ALDH inhibitor, as a negative control. The ALDH-positive population was established, according to the manufacturer's instructions and was evaluated using 30,000 cells.

### Proteomics and Ingenuity Pathway Analysis (IPA)

Samples were submitted to the CRUK Proteomics Core Facility, for label-free proteomic analysis. Proteomics and statistical analyses were carried out on a fee-for-service basis by Smith and his colleagues, at the Proteomics Core Facility at the Cancer Research UK Manchester Institute, University of Manchester. Briefly, stably transduced MCF7 cells were cultured in complete media at a density of 1.8 × 10^6^ in 10 cm dish. The day after plating, the media was changed to DMEM with 10% NuSerum, Pen-Strep and Glutamax. After 24 h, 70–75% of confluence was reached. Then, cells were washed twice with PBS and RIPA lysis buffer was added without protease inhibitors to detach the cells. The lysates were collected into pre-cooled tubes and kept on ice for 10 min. After centrifugation, the supernatants were collected and the samples were flash-frozen using liquid nitrogen. Previously, a small aliquot was removed for protein quantification. Samples were kept at −80°C until further analysis. Samples were subjected to proteomics following a protocol previously described (27). Briefly, cell lysates were prepared for trypsin digestion by sequential reduction of disulfide bonds with TCEP and alkylation with MMTS. Then, the peptides were extracted and prepared for LC-MS/MS. All LC-MS/MS analyses were performed on an LTQ Orbitrap XL mass spectrometer (ThermoScientific) coupled to an Ultimate 3000 RSLCnano system (ThermoScientific). Xcalibur raw data files acquired on the LTQ-Orbitrap XL were directly imported into Progenesis LCMS software (Waters Corp.) for peak detection and alignment. Data were analyzed using the Mascot search engine. Five replicates were analyzed for each sample type (N = 5). Statistical analyses were performed using ANOVA and a 1.5-fold-change in protein levels, with a *p* < 0.05 were considered significant. The molecular function and biological pathways of the differentially expressed proteins were performed by the unbiased interrogation and analysis of proteomic data sets using IPA (Ingenuity systems, http://www.ingenuity.com). IPA assists with data interpretation, via the grouping of differentially expressed genes or proteins into known functions and pathways. Pathways with a z score of > +2 were considered as significantly activated, while pathways with a z score of <-2 were considered as significantly inhibited.

### Statistical Analysis

Data is represented as the mean ± standard error of the mean (SEM), taken over ≥3 independent experiments, with ≥3 technical replicates per experiment, unless stated otherwise. Statistical significance was measured, using the Student *t*-test. A *p* < 0.05 was considered statistically significant.

## Results

Cancer stem cells (CSCs) are characterized by elevated mitochondrial biogenesis and metabolism (2). However, mitochondrial function is also largely dependent on a well-regulated balance between mitochondrial fusion and fission dynamics (19, 23). In fact, aberrantly activated fission results in mitochondrial fragmentation, which is associated to mitochondrial dysfunction.

Here, we interrogated how unopposed mitochondrial fission may promote alterations in mitochondrial biology and function, leading to inhibition of CSCs propagation in breast cancer.

### MFF Inhibits Mitochondrial Biogenesis

**Figure 1:**
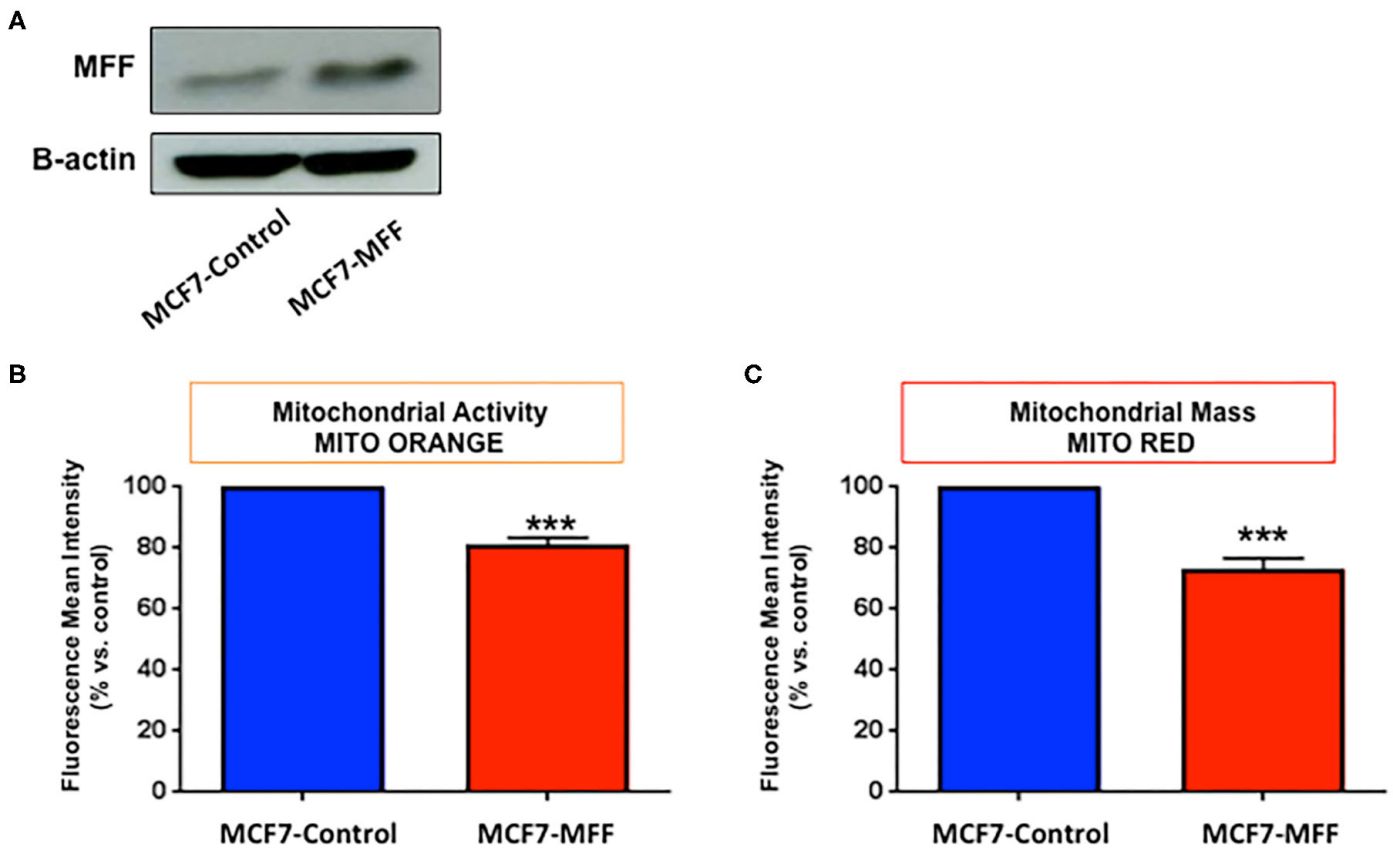
Mitochondrial fission factor (MFF) decreases mitochondrial activity and mass. **(A)** Evaluation of MFF overexpression. MCF7 cells, stably transduced with a lentiviral vector encoding for mitochondrial fission factor (MCF-MFF) or the empty-vector (MCF-7 Control), were subjected to protein extraction and immunoblotted for MFF. β-actin is shown as equal loading control. **(B,C)** MFF overexpression decreases mitochondrial activity and mass. Stably transduced MCF7 cells harboring MFF (MCF-MFF) and the respective empty-vector (MCF-7 Control) were seeded for 24 h and then mitochondrial activity and mitochondrial mass were quantitated by FACS analysis using the probes MitoTracker Orange **(B)** and MitoTracker Deep-Red **(C)**. At least four replicates were performed in each experiment. Results are the average of the mean of three independent experiments and are expressed as percentages normalized to the control ± SEM. ***p < 0.001.

In order to investigate the role of MFF in the regulation of mitochondrial activity in breast cancer cells, we generated an isogenic MCF7 cell line harboring MFF (MCF7-MFF), together with a matched isogenic cell line harboring the empty vector, which served as a control (MCF7-Control). After verifying MFF-overexpression by Western blotting (Figure 1A), the newly generated cell lines were subjected to functional phenotypic characterization. As a first step, cells were analyzed by FACS analysis using MitoTracker Deep-Red-FM, as a probe to estimate mitochondrial mass. As shown in Figure 1C, mitochondrial content was reduced by 30% in MCF7-MFF cells. A similar trend was observed for the evaluation of mitochondrial activity by FACS analysis, using the probe Mito-Orange (Figure 1B), suggesting an overall impairment in mitochondrial content and function in the presence of MFF-overexpression.

### MFF Inhibits Breast Cancer Cell Metabolism

**Figure 2:**
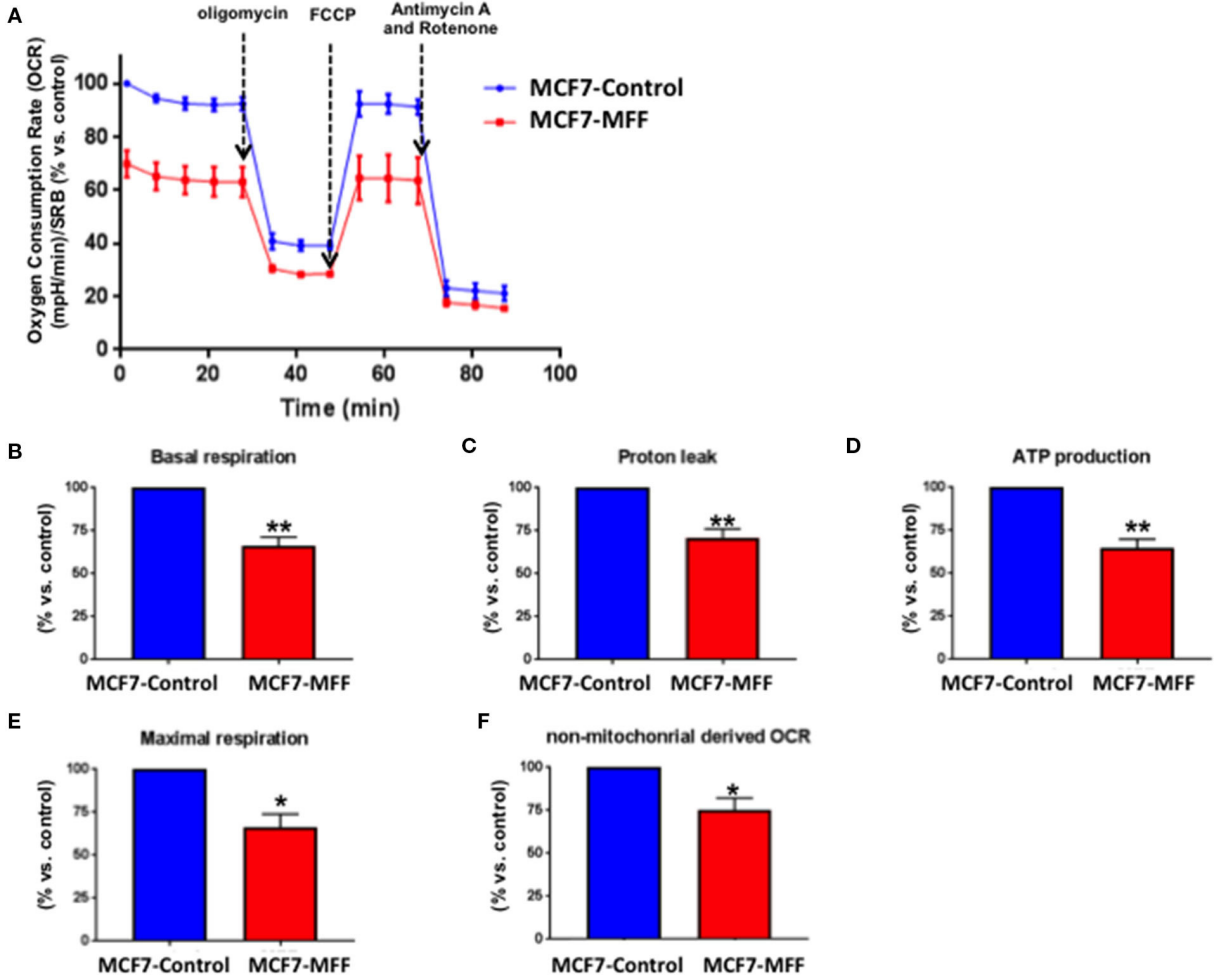
Mitochondrial fission factor (MFF) reduces mitochondrial respiration. The metabolic profile of stably transduced MCF7 cells harboring MFF (MCF-MFF) and the respective empty-vector (MCF-7 Control) was examined using the Seahorse XFe96 analyzer. **(A)** Oxygen consumption rate (OCR) is significantly reduced in cells transduced with MFF as compared to control cells. **(B–F)** Significant reductions in respiration (basal and maximal), proton leak, ATP levels and non-mitochondrial derived OCR were observed in MCF7 cells transduced with MFF as compared to the control cells. At least six replicates were performed in each experiment. Results are the average of the mean of three independent experiments and are expressed as percentages normalized to the control ± SEM. *p < 0.05, **p < 0.01.

**Figure 3:**
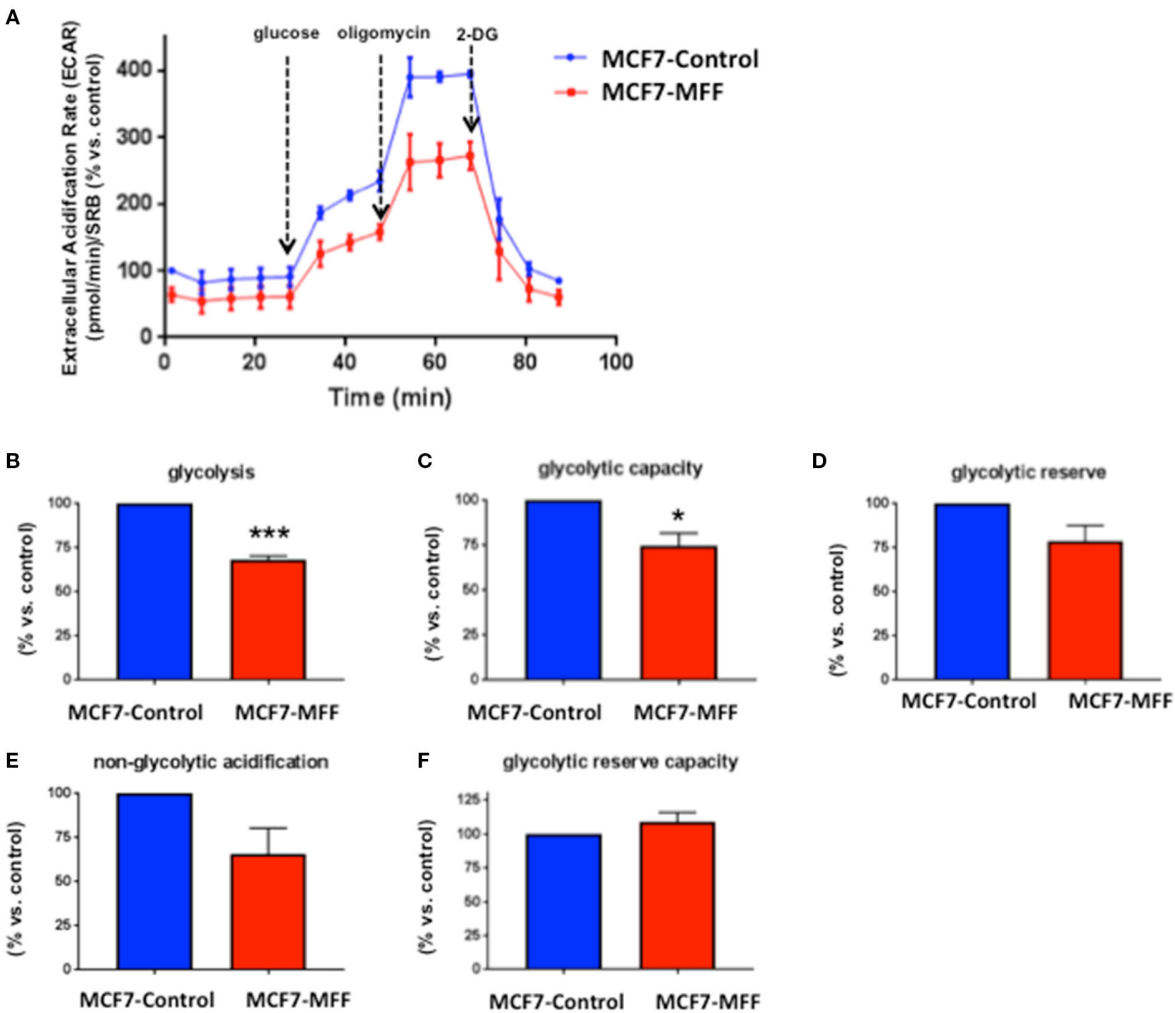
Mitochondrial fission factor (MFF) reduces glycolysis. The metabolic profile of stably transduced MCF7 cells harboring MFF (MCF-MFF) and the respective empty-vector (MCF-7 Control) was examined using the Seahorse XFe96 analyzer. **(A)** Extracellular acidification rate (ECAR) is significantly reduced in cells transduced with MFF as compared to control cells. **(B,C)** Significant reduction in glycolysis and glycolytic capacity were observed in MCF7 cells transduced with MFF as compared to control cells, without significant changes in glycolytic reserve **(D)**, non-glycolytic acidification **(E)**, and glycolytic reserve capacity **(F)**. At least six replicates were performed in each experiment. Results are the average of the mean of three independent experiments and are expressed as percentages normalized to the control ± SEM. *p < 0.05, ***p < 0.001.

Data shown above immediately suggest that MFF may interfere with mitochondrial oxidative metabolism. To test this hypothesis, we directly evaluated metabolic flux using the Seahorse XFe96 and we found that Oxygen Consumption Rates (OCR) were significantly reduced in MCF7-MFF cells (Figure 2). More specifically, basal respiration and maximal respiration were reduced by nearly 40% (Figures 2B,E); accordingly, ATP levels were also depleted (Figure 2D). Notably, the analysis of Extracellular Acidification Rates (ECR) demonstrated that also the glycolytic pathway is inhibited in MCF7-MFF cells (Figure 3). In particular, a nearly 40 % decrease in glycolysis and glycolytic capacity (Figures 3B,C) were observed; an inhibitory, though not significant, trend was also observed for glycolytic reserve and non-glycolytic acidification (Figures 3D,E). Taken together, these data indicate that MFF over-expression negatively affects the metabolic cell machinery, by interfering with both oxidative phosphorylation and glycolysis in breast cancer cells.

### MFF Inhibits Breast CSC Activity

**Figure 4:**
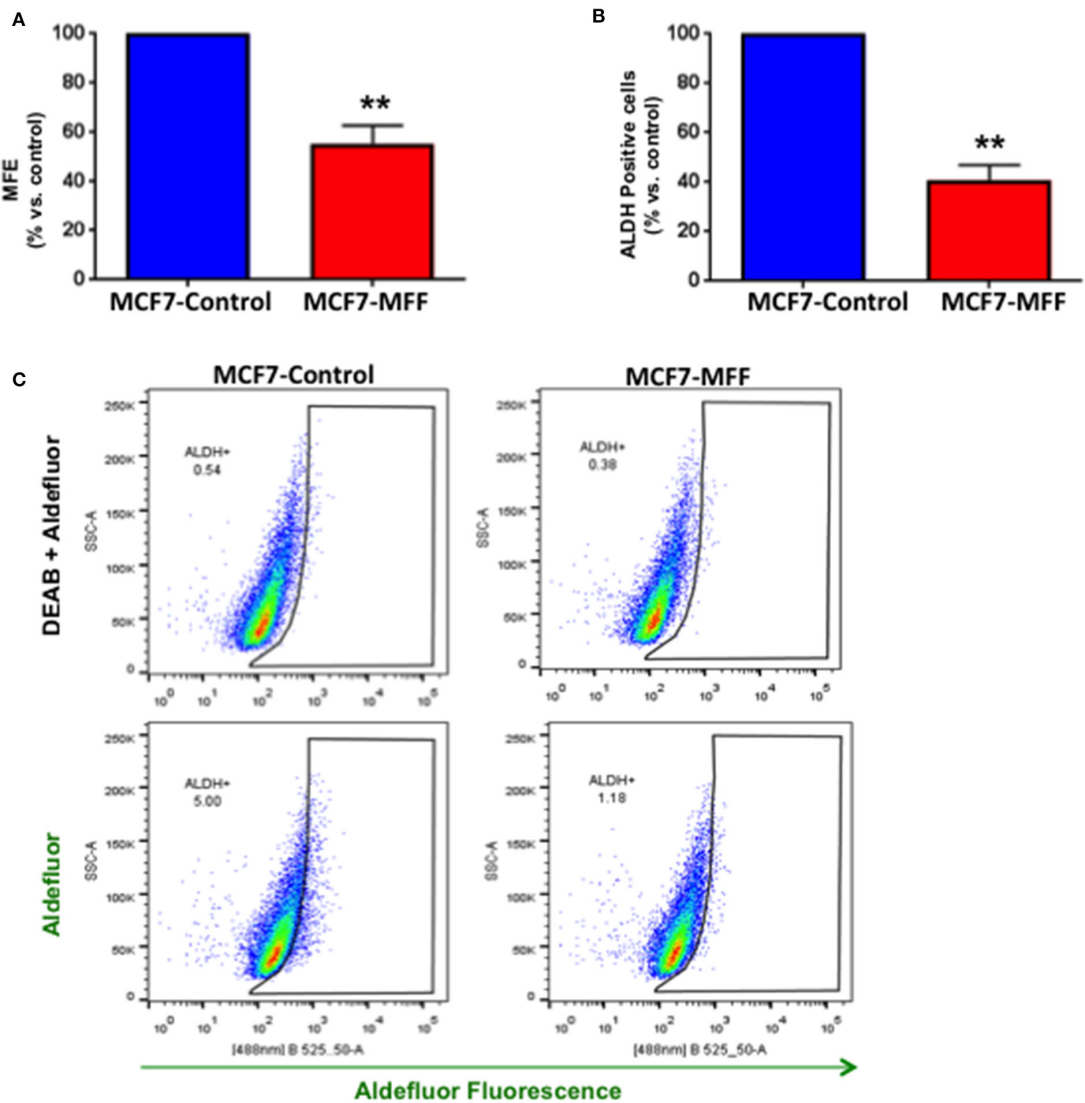
Mitochondrial fission factor (MFF) inhibits 3D-spheroid formation and ALDH activity. **(A)** Evaluation of mammosphere formation efficiency (MFE) in stably transduced MCF7 cells harboring the mitochondrial fission factor (MCF7-MFF) and the respective empty-vector (MCF-7 Control), which were seeded on low-attachment plates for 5 days. Under these conditions, MCF7-MFF cells show a reduction by 45% in the mammosphere forming capacity as compared to MCF7-Control cells. **(B)** Evaluation of ALDEFLUOR activity, an independent marker of CSCs in MCF7 cells harboring MFF (MCF7-MFF) and the respective empty vector control (MCF-Control). Each sample was normalized using diethylaminobenzaldehyde (DEAB), a specific ALDH inhibitor, as negative control. **(C)** The tracing of representative samples is shown. Results are the average of the mean of three independent experiments performed in triplicate and are expressed as percentages normalized to the control ± SEM. **p < 0.01.

We previously established that within the heterogeneous tumor mass, cancer cells exhibiting stemness features are also characterized by an elevated level of mitochondrial function (5, 6, 28). On the other hand, strategies aimed at targeting mitochondria have proven to be beneficial in halting CSCs, both in pre-clinical and clinical studies (9, 12, 29, 30). Based on these observations, we investigated whether aberrant mitochondrial fission may affect CSC propagation, together with the impairment of mitochondrial function. For this purpose, we used the 3D tumor-sphere formation assay as readout for CSCs activity. Figure 4A shows that mammosphere formation is inhibited by nearly 50% in MCF7-MFF cells. Likewise, ALDH activity, a surrogate marker of stemness, was reduced by >60% in MCF7-MFF cells (Figures 4B,C). These results clearly indicate that MFF over-expression hampers breast CSCs propagation.

### Proteomic Profiling Reveals the MFF-Dependent Metabolic, Signaling, and Biological Landscape

Dysfunctional mitochondria activate a retrograde signaling network that shapes the nuclear transcriptomic program toward the regulation of cell morphology and function, for cells to response to disruption of energy metabolism (31). Adding to this, several lines of evidence indicate that mitochondrial dynamics might be involved in the regulation of stress signaling (14). Hence, we sought to depict the metabolic and signaling network landscape of MFF over-expressing breast cancer cells by using an “omics” approach. More specifically, unbiased proteomic analysis was performed in MCF7-MFF as well as in the matched MCF7-Control counterpart. Thereafter, the software Ingenuity Pathway was used to: (i) classify and group the proteins differentially regulated in response to MFF overexpression; (ii) predict how the upstream regulators may cause downstream phenotypic or functional changes in response to MFF overexpression.

### Metabolomic Signature

**Figure 5:**
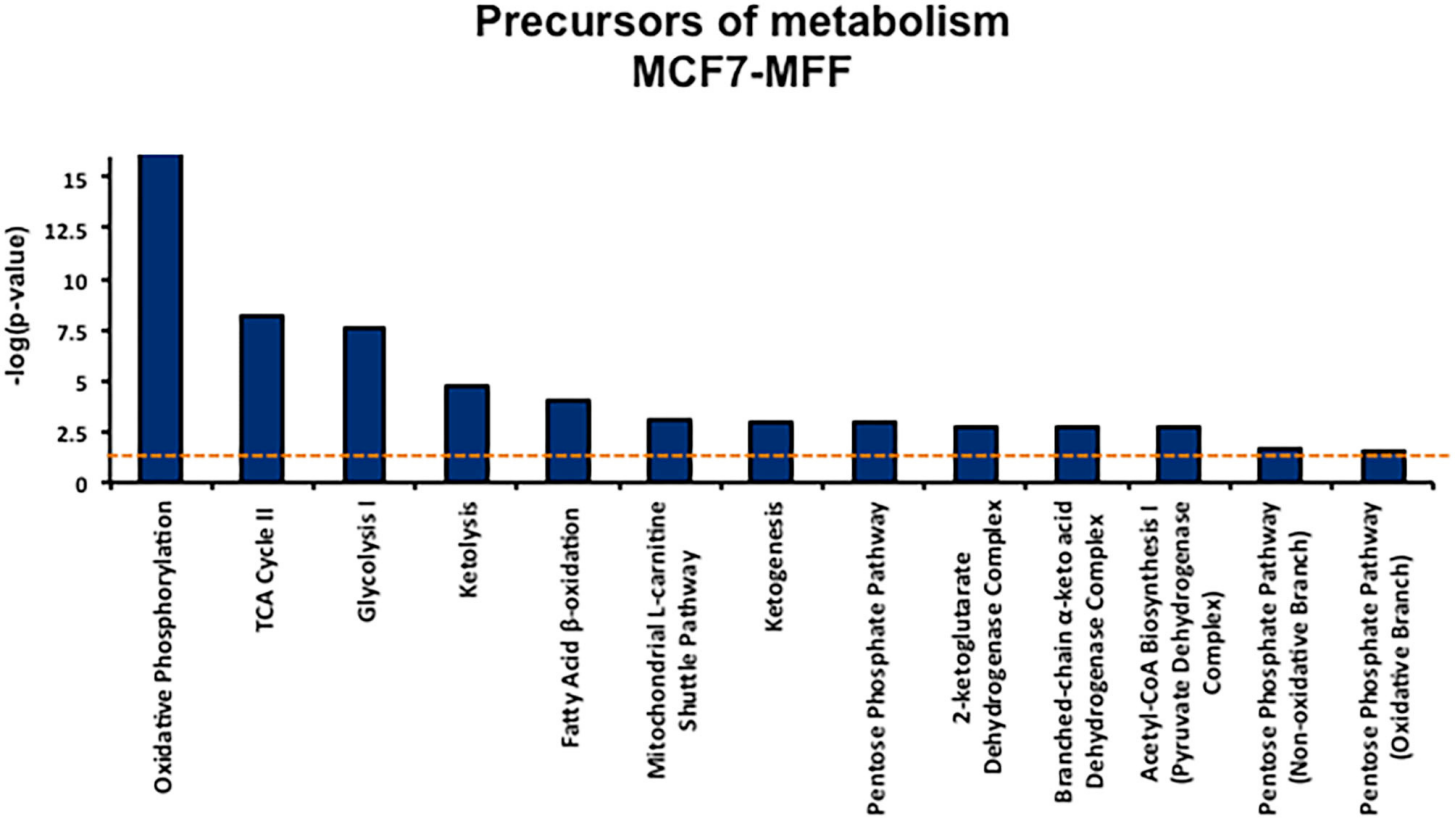
Precursors of metabolism list. Precursor of metabolism list enriched by the proteins significantly regulated in MCF7 harboring mitochondrial fission factor (MFF) compared to MCF7 Control cells. The list has been generated by Ingenuity Pathway Analysis (IPA). The *p*-value for each pathway is represented by the height of the bars and is expressed as −1 times the log of the *p*-value. The orange dotted line represents the threshold of *p* = 0.05 as calculated by Fischer's test and denotes the cut-off for significance.

**Figure 6:**
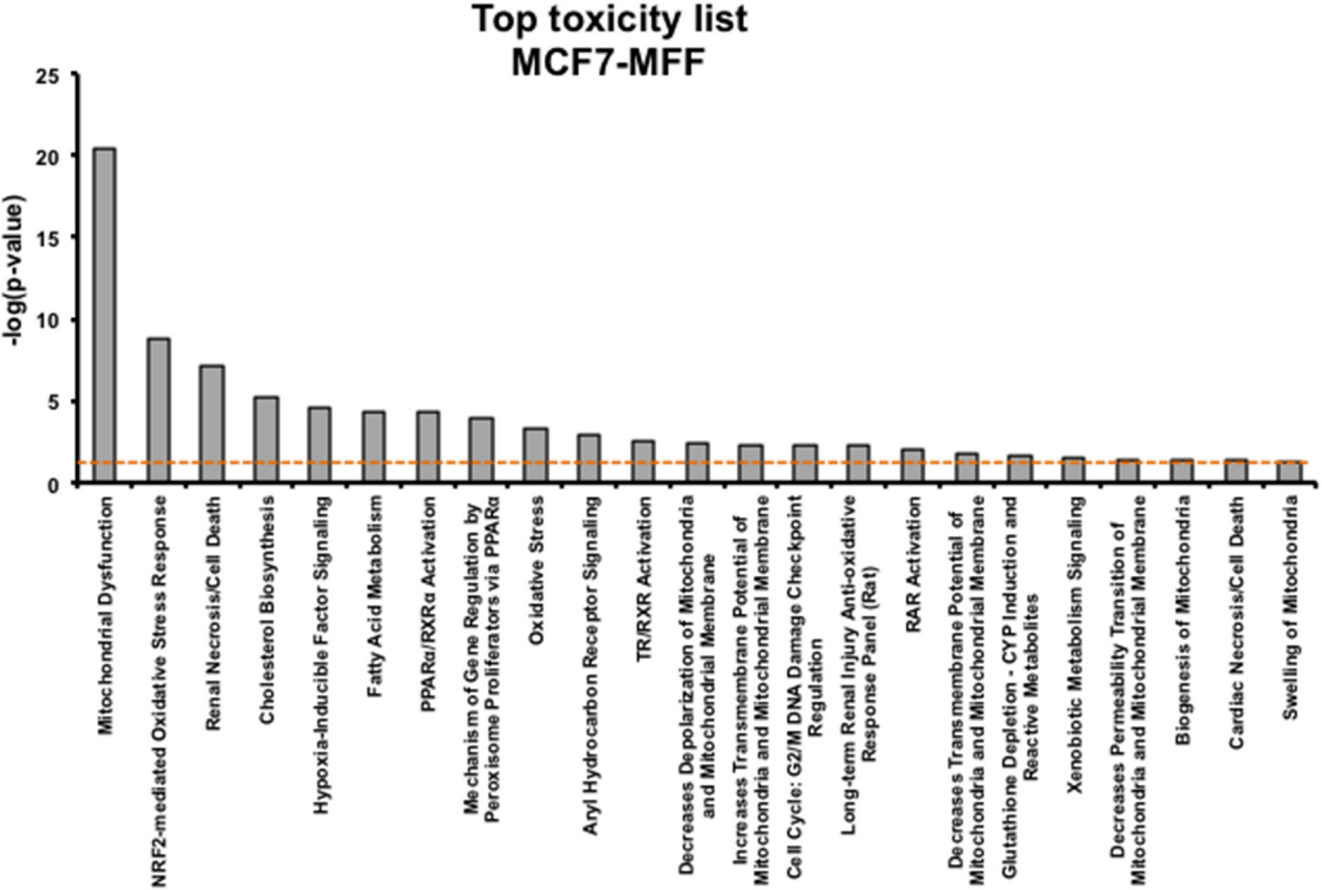
Toxicological Pathways and Functions significantly regulated in MCF7 cells overexpressing the mitochondrial fission factor (MFF). Toxicity list enriched by the proteins significantly regulated in MCF7 harboring mitochondrial fission factor (MFF) has been generated by Ingenuity Pathway Analysis (IPA). The *p*-value for each pathway is represented by the height of the bars and is expressed as −1 times the log of the *p*-value. The orange dotted line represents the threshold of *p* = 0.05 as calculated by Fischer's test and denotes the cut-off for significance.

Results, shown in Tables 1–5, indicate that increased expression of MFF fosters a transition toward a metabolically quiescent cell phenotype. In particular, proteins involved in mitochondrial biogenesis (Table 1), Oxidative Phosphorylation (Table 2) and TCA cycle (Table 3) are the most strongly down-regulated in MCF7-MFF, as compared to MCF-Control cells. Furthermore, inhibition of proteins involved in the glycolytic and the pentose phosphate pathways is observed in MCF7-MFF cells (Tables 4, 5). Likewise, a higher accumulation of proteins involved in the metabolism of metabolic precursors mainly for Oxidative phosphorylation, TCA cycle and Glycolysis is detected in MCF7-MFF cells, suggesting that the metabolic machinery is inhibited in the presence of enhanced mitochondrial fragmentation (Figure 5). Also, it appears that MFF over-expression triggers the up-regulation of a “toxicity network” of proteins, mainly related to cell metabolism (Figure 6). The most strongly up-regulated “toxicity” proteins are classified as regulators of mitochondrial dysfunction, together with oxidative stress response and fatty acid metabolism (Figure 6). Also, a “toxicity network” of proteins involved in the regulation of mitochondrial membrane potential and hypoxia signaling are engaged as relevant effectors in MCF7-MFF cells (Figure 6).

**Table 1:** Significant changes in protein levels associated with mitochondrial biogenesis in MCF7 cells over-expressing mitochondrial fission factor (MFF).

**Symbol**	**Gene name**	**Fold change**
**Mitochondrial biogenesis**		
MFN2	Mitofusin 2	**−31.184**
MTERF1	Mitochondrial transcription termination factor 1	**−5.38**
MTFR1	Mitochondrial fission regulator 1	**−9.616**
TFAM	Transcription factor A, mitochondrial	**1.519**
TIMM50	Translocase of inner mitochondrial membrane 50	**−83.102**
TIMM10B	Translocase of inner mitochondrial membrane 10 homolog B (yeast)	**−157.576**
TIMM23B	Translocase of inner mitochondrial membrane 23 homolog B	**1.56**
TIMM8B	Translocase of inner mitochondrial membrane 8 homolog B	**−5.662**
TIMM13	Translocase of inner mitochondrial membrane 13	**−2.266**
TOMM34	Translocase of outer mitochondrial membrane 34	**−5.272**
TSFM	Ts translation elongation factor, mitochondrial	**1.635**
YY1	YY1 transcription factor	**Infinity**
SIRT6	Sirtuin 6	**−8.746**

*Fold change of proteins detected in MCF7-MFF vs. MCF7-Control cells. Red: Up-regulated proteins; Green: down-regulated proteins. Proteomics was performed as described in Materials and Methods. Statistical analyses were performed using ANOVA and 1.5-fold-changes in proteins with a p < 0.05 were considered. Dataset containing proteins with significant altered expression profile were imported into the Ingenuity Pathway Analyses (IPA) software, which groups the differentially expressed proteins into known functions and pathways*.

**Table 2:** Significant changes in protein levels associated with oxidative phosphorylation in MCF7 cells over-expressing the mitochondrial fission factor (MFF).

**Symbol**	**Gene name**	**Fold change**
**Oxidative phosphorylation**		
ATP5A1	ATP synthase, H+ transporting, mitochondrial F1 complex, alpha subunit 1, cardiac muscle	**9.846**
ATP5B	ATP synthase, H+ transporting, mitochondrial F1 complex, beta polypeptide	**1781.432**
ATP5C1	ATP synthase, H+ transporting, mitochondrial F1 complex, gamma polypeptide 1	**−2.919**
ATP5F1	ATP synthase, H+ transporting, mitochondrial Fo complex subunit B1	**2.216**
ATP5H	ATP synthase, H+ transporting, mitochondrial Fo complex subunit D	**1.623**
ATP5I	ATP synthase, H+ transporting, mitochondrial Fo complex subunit E	**1.736**
ATP5J	ATP synthase, H+ transporting, mitochondrial Fo complex subunit F6	**1.673**
ATP5J2	ATP synthase, H+ transporting, mitochondrial Fo complex subunit F2	**−2.37**
ATP5L	ATP synthase, H+ transporting, mitochondrial Fo complex subunit G	**−15.581**
ATP5O	ATP synthase, H+ transporting, mitochondrial F1 complex, O subunit	**−601.813**
COX4I1	Cytochrome c oxidase subunit 4I1	**1.669**
COX4I2	Cytochrome c oxidase subunit 4I2	**−137.623**
COX5A	Cytochrome c oxidase subunit 5A	**1.913**
COX5B	Cytochrome c oxidase subunit 5B	**2.364**
COX6B1	Cytochrome c oxidase subunit 6B1	**1.87**
COX6C	Cytochrome c oxidase subunit 6C	**2.38**
COX7A2	Cytochrome c oxidase subunit 7A2	**−12.37**
COX7A2L	Cytochrome c oxidase subunit 7A2 like	**1.617**
CYB5A	Cytochrome b5 type A	**1.802**
CYC1	Cytochrome c1	**16.811**
CYCS	Cytochrome c, somatic	**1.821**
MT-ATP6	ATP synthase F0 subunit 6	**−280.464**
MT-CO2	Cytochrome c oxidase subunit II	**4.759**
MT-ND1	NADH dehydrogenase, subunit 1 (complex I)	**11.786**
MT-ND2	Mitochondrially encoded NADH dehydrogenase 2	**−7.366**
MT-ND5	NADH dehydrogenase, subunit 5 (complex I)	**−8.753**
NDUFA2	NADH:ubiquinone oxidoreductase subunit A2	**1.51**
NDUFA4	NDUFA4, mitochondrial complex associated	**2.201**
NDUFA5	NADH:ubiquinone oxidoreductase subunit A5	**1.527**
NDUFA6	NADH:ubiquinone oxidoreductase subunit A6	**−112.999**
NDUFA8	NADH:ubiquinone oxidoreductase subunit A8	**45.057**
NDUFA9	NADH:ubiquinone oxidoreductase subunit A9	**3.523**
NDUFA10	NADH:ubiquinone oxidoreductase subunit A10	**10.495**
NDUFA12	NADH:ubiquinone oxidoreductase subunit A12	**−60.713**
NDUFA13	NADH:ubiquinone oxidoreductase subunit A13	**1.767**
NDUFAB1	NADH:ubiquinone oxidoreductase subunit AB1	**−3.356**
NDUFB3	NADH:ubiquinone oxidoreductase subunit B3	**1.558**
NDUFB4	NADH:ubiquinone oxidoreductase subunit B4	**2.211**
NDUFB5	NADH:ubiquinone oxidoreductase subunit B5	**−338.956**
NDUFB6	NADH:ubiquinone oxidoreductase subunit B6	**2.247**
NDUFB7	NADH:ubiquinone oxidoreductase subunit B7	**−20.943**
NDUFB9	NADH:ubiquinone oxidoreductase subunit B9	**2.454**
NDUFB10	NADH:ubiquinone oxidoreductase subunit B10	**1.609**
NDUFS1	NADH:ubiquinone oxidoreductase core subunit S1	**−77.83**
NDUFS2	NADH:ubiquinone oxidoreductase core subunit S2	**−22.623**
NDUFS3	NADH:ubiquinone oxidoreductase core subunit S3	**−21.085**
NDUFS5	NADH:ubiquinone oxidoreductase subunit S5	**1.686**
NDUFS6	NADH:ubiquinone oxidoreductase subunit S6	**1.977**
NDUFS7	NADH:ubiquinone oxidoreductase core subunit S7	**−69.072**
NDUFS8	NADH:ubiquinone oxidoreductase core subunit S8	**−45.818**
NDUFV1	NADH:ubiquinone oxidoreductase core subunit V1	**1.552**
NDUFV2	NADH:ubiquinone oxidoreductase core subunit V2	**−5.91**
SDHA	Succinate dehydrogenase complex flavoprotein subunit A	**1.617**
SDHB	Succinate dehydrogenase complex iron sulfur subunit B	**2.018**
UQCR10	Ubiquinol-cytochrome c reductase, complex III subunit X	**2.354**
UQCR11	Ubiquinol-cytochrome c reductase, complex III subunit XI	**1.501**
UQCRB	Ubiquinol-cytochrome c reductase binding protein	**1.534**
UQCRC1	Ubiquinol-cytochrome c reductase core protein I	**−4.993**
UQCRC2	Ubiquinol-cytochrome c reductase core protein II	**373.541**
UQCRH	Ubiquinol-cytochrome c reductase hinge protein	**−40.845**
UQCRQ	Ubiquinol-cytochrome c reductase complex III subunit VII	**9.76**

*Fold change of proteins detected in MCF7-MFF vs. MCF7-Control cells. Red: Up-regulated proteins; Green: Down-regulated proteins. Proteomics was performed as described in Materials and Methods. Statistical analyses were performed using ANOVA and 1.5-fold-changes in proteins with a p < 0.05 were considered. Dataset containing proteins with significant altered expression profile were imported into the Ingenuity Pathway Analyses (IPA) software, which groups the differentially expressed proteins into known functions and pathways*.

**Table 3:** Significant changes in protein levels associated with the tricarboxylic acid cycle in MCF7 cells over-expressing mitochondrial fission factor (MFF).

**Symbol**	**Gene name**	**Fold change**
**TCA cycle**		
ACO1	Aconitase 1	**−33.921**
ACO2	Aconitase 2	**−14.648**
CS	Citrate synthase	**−34.112**
DHTKD1	Dehydrogenase E1 and transketolase domain containing 1	**−3.207**
DLD	Dihydrolipoamide dehydrogenase	**3.814**
DLST	Dihydrolipoamide S-succinyltransferase	**−3.202**
FH	Fumarate hydratase	**14.079**
IDH1	Isocitrate dehydrogenase [NADP(+)] 1, cytosolic	**4.014**
IDH2	Isocitrate dehydrogenase [NADP(+)] 2, mitochondrial	**−176.206**
IDH3A	Isocitrate dehydrogenase 3 [NAD(+)] alpha	**−17.146**
IDH3B	Isocitrate dehydrogenase 3 [NAD(+)] beta	**1.752**
IDH3G	Isocitrate dehydrogenase 3 [NAD(+)] gamma	**3.159**
MDH1	Malate dehydrogenase 1	**24.472**
MDH2	Malate dehydrogenase 2	**−211.122**
OGDH	Oxoglutarate dehydrogenase	**−47.17**
OGDHL	Oxoglutarate dehydrogenase-like	**4.76**
SDHA	Succinate dehydrogenase complex flavoprotein subunit A	**1.617**
SDHB	Succinate dehydrogenase complex iron sulfur subunit B	**2.018**
SUCLA2	Succinate-CoA ligase ADP-forming beta subunit	**3.127**
SUCLG1	Succinate-CoA ligase alpha subunit	**6.425**

*Fold change of proteins detected in MCF7-MFF vs. MCF7-Control cells. Red: Up-regulated proteins; Green: Down-regulated proteins. Proteomics was performed as described in Materials and Methods. Statistical analyses were performed using ANOVA and 1.5-fold-changes in proteins with a p < 0.05 were considered. Dataset containing proteins with significant altered expression profile were imported into the Ingenuity Pathway Analyses (IPA) software, which groups the differentially expressed proteins into known functions and pathways*.

**Table 4:** Significant changes in protein levels associated with glycolysis in MCF7 cells over-expressing mitochondrial fission factor (MFF).

**Symbol**	**Gene name**	**Fold change**
**Glycolysis**		
GLUT1	Facilitated glucose transporter member 1	**−27.22**
HXK1	Hexokinase 1	**−17.96**
HXK2	Hexokinase 2	**–Infinity**
HXK3	Hexokinase 3	**1.755**
ALDOA	Aldolase, fructose-bisphosphate A	**−63.01**
ALDOC	Aldolase, fructose-bisphosphate C	**3.304**
ENO1	Enolase 1	**−353.572**
ENO2	Enolase 2	**−14.088**
ENO3	Enolase 3	**4.658**
FBP1	Fructose-bisphosphatase 1	**−5.137**
GAPDH	Glyceraldehyde-3-phosphate dehydrogenase	**37.238**
GPI	Glucose-6-phosphate isomerase	**3.486**
PFKM	Phosphofructokinase, muscle	**−5.855**
PFKP	Phosphofructokinase, platelet	**–Infinity**
PGAM1	Phosphoglycerate mutase 1	**3.86**
PGAM4	Phosphoglycerate mutase family member 4	**1.668**
PGK1	Phosphoglycerate kinase 1	**250.959**
PGK2	Phosphoglycerate kinase 2	**−28.097**
PKLR	Pyruvate kinase, liver and RBC	**−11.843**
PKM	Pyruvate kinase, muscle	**−92.492**
TPI1	Triosephosphate isomerase 1	**−44.171**
LDHA	Lactate dehydrogenase A	**2.82**
LDHA6LB	Lactate dehydrogenase A like 6B	**−58.176**

*Fold change of proteins detected in MCF7-MFF vs. MCF7-Control cells. Red: Up-regulated proteins; Green: Down-regulated proteins. Proteomics was performed as described in Materials and Methods. Statistical analyses were performed using ANOVA and 1.5-fold-changes in proteins with a p < 0.05 were considered. Dataset containing proteins with significant altered expression profile were imported into the Ingenuity Pathway Analyses (IPA) software, which groups the differentially expressed proteins into known functions and pathways*.

**Table 5:** Significant changes in protein levels associated with the pentose phosphate pathway in MCF7 cells overexpressing mitochondrial fission factor (MFF).

**Symbol**	**Gene name**	**Fold change**
**Pentose phosphate pathway**		
G6PD	Glucose-6-phosphate dehydrogenase	**−136.908**
PGD	Phosphogluconate dehydrogenase	**4.903**
PGLS	6-phosphogluconolactonase	**1.776**
RPE	Ribulose-5-phosphate-3-epimerase	**2.25**
RPIA	Ribose 5-phosphate isomerase A	**−11.28**
TALDO1	Transaldolase 1	**−6.741**
TKT	Transketolase	**−82.993**

*Fold change of proteins detected in MCF7-MFF vs. MCF7-Control cells. Red: Up-regulated proteins; Green: Down-regulated proteins. Proteomics was performed as described in Materials and Methods. Statistical analyses were performed using ANOVA and 1.5-fold-changes in proteins with a p < 0.05 were considered. Dataset containing proteins with significant altered expression profile were imported into the Ingenuity Pathway Analyses (IPA) software, which groups the differentially expressed proteins into known functions and pathways*.

### Signaling Pathways and Predicted Biological Responses

**Figure 7:**
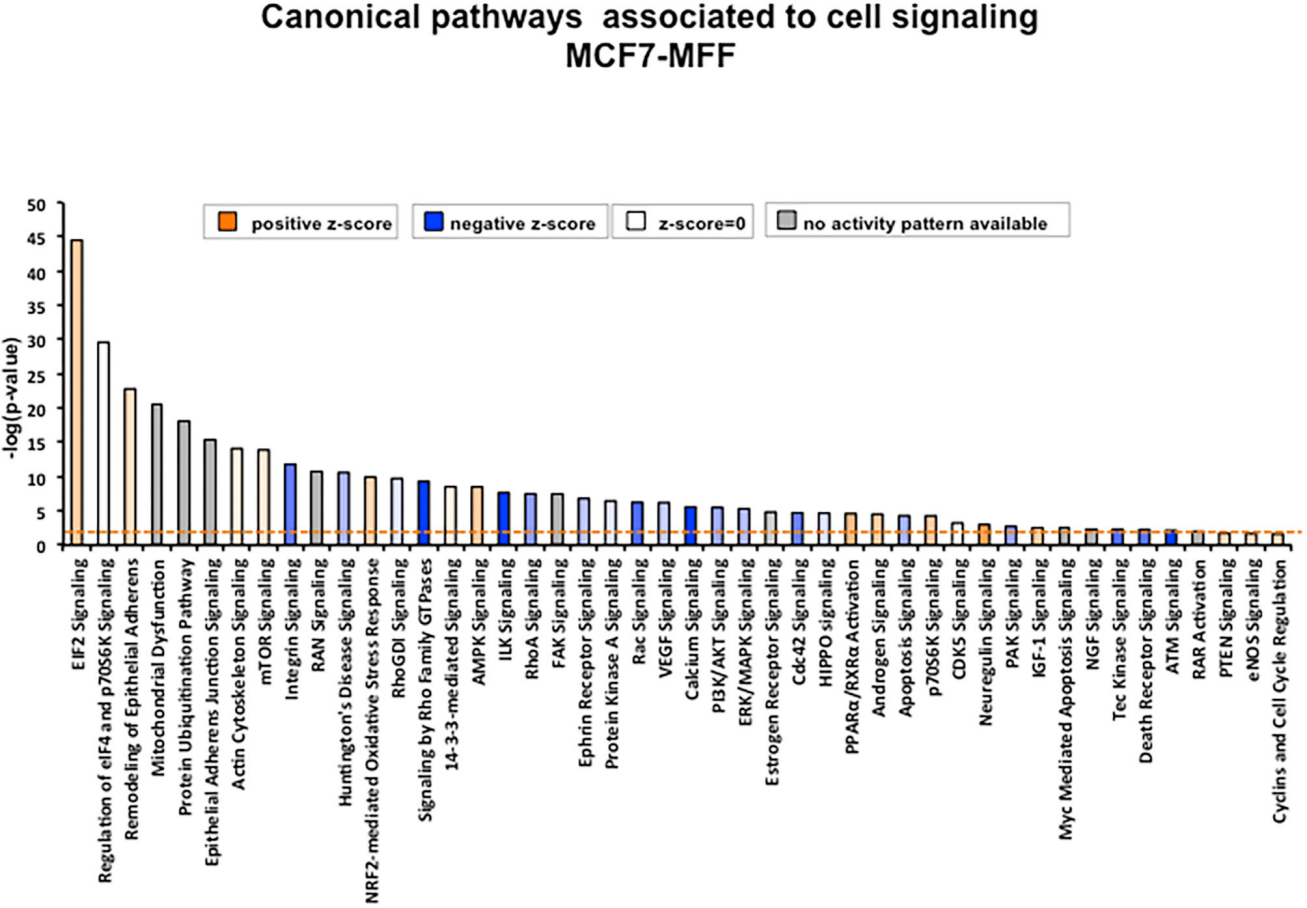
Canonical pathways enriched in proteins differentially regulated in MCF7 cells overexpressing the mitochondrial fission factor (MFF). Significant canonical pathways associated to cell signaling were generated by Ingenuity Pathway Analysis (IPA). X axis represents pathways and Y axis represents –log (*p*-value) calculated with Fisher's exact test. A *p*-value threshold of 0.05 is applied and is represented by the orange dotted line.

**Figure 8:**
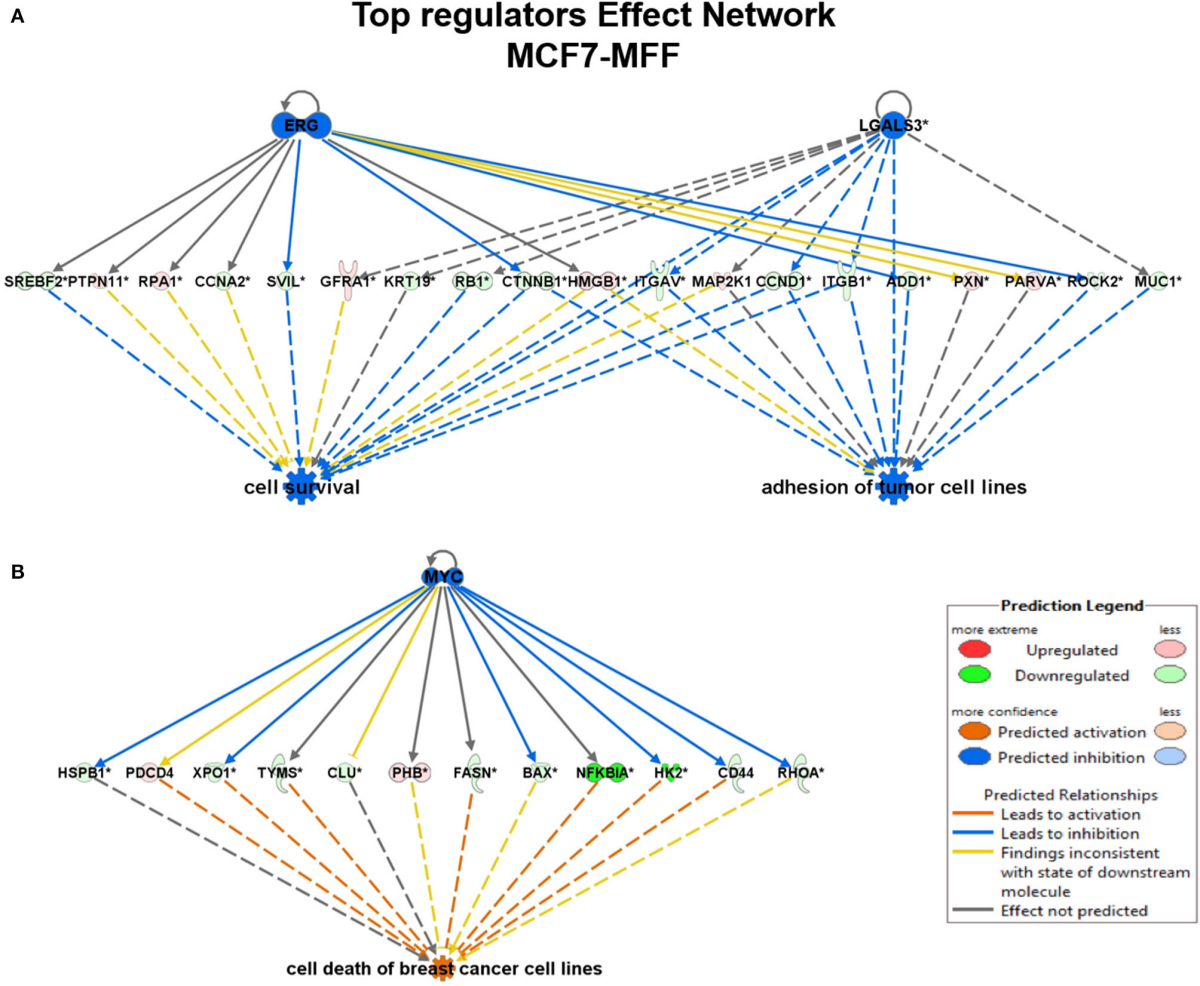
Regulator effect networks generated by Ingenuity Pathway Analysis (IPA) in MCF7 over-expressed mitochondrial fission factor (MFF). IPA analysis generated several regulator effect networks in MCF7 cells over-expressing mitochondrial fission factor (MFF). Both upstream regulators and downstream cellular responses were identified in relation to certain proteins significantly altered in our dataset. Upstream regulators are shown at the top while functions and phenotypes are displayed at the bottom. Significantly regulated proteins connecting the upper and lower panel are displayed in the middle. Red and green colors represent significant up/down-regulation, respectively, with the intensity of the color reflecting the degree of change. Orange color predicts an overall activation of the pathway while blue indicates a prediction of an overall decrease based on z-score value. Inconsistent relationships are represented as yellow line. Note that in MCF7 harboring MFF some of the proteins in our dataset have been predicted to be inhibited by the upstream regulators ERG, LGALS3, and MYC leading to an overall inhibition (blue) of cell survival and/or activation (orange) of cell death. **(A)** Cell survival and adhesion; **(B)** Cell death.

To interrogate how MFF over-expression would trigger the activation of specific intracellular signaling cascades, which are predicted to control distinct biological responses, a dataset containing proteins with significant altered expression profile was imported into the IPA tool. As shown in Figure 7, the most strongly differentially activated pathways in MCF7-MFF cells are predicted to regulate EI2F signaling, as well as epithelial adherens junctions and the cytoskeleton. Accordingly, down-regulation of proteins involved in Epithelial Mesenchymal Transition (EMT), Extracellular Matrix (ECM) and cytoskeleton remodeling is detected in MCF-MFF cells (Table 6). In addition, proteins involved in metastasis formation (MTA1 and MTA2), oxidative stress signaling (CAT, SOD2, TXNRD1, GLRX3, GSR, TXNL1, TXNRD2, TXNRD3), TGFß pathway (TGFB1, TGFBR3, TGFIF2lX, STAT1, STAT3) and TNF-alpha (TNFRSF12A, TRAF2) were all dramatically down-regulated in MCF7-MFF cells (Table 7). In order to further characterize the signaling landscape of MCF7-MFF cells, we performed a Regulator-Effects analysis, which integrates and merges together Upstream Regulator Networks with Downstream Effect Networks, thereby deriving how predicted regulators might impact biological processes. As shown in Figure 8, inhibition of cell adhesion and survival (A), and activation of cell death response (B) are the main biological events associated with MFF over-expression. The identification of the signaling mediators potentially linking upstream mediators with down-stream effects is also shown. Taken together, these data suggest that aberrant mitochondrial fragmentation may halt breast cancer cell survival and activate cell death programs by regulating a number of effectors also involved in ECM remodeling, the oxidative stress response, and mitochondrial metabolic function.

**Table 6:** Significant changes in protein levels associated with the epithelial-mesenchymal transition (EMT), extracellular matrix and cytoskeleton in MCF7 cells over-expressing mitochondrial fission factor (MFF).

**Symbol**	**Gene name**	**Fold change**
**Epithelial-mesenchymal transition, ECM and cytoskeleton proteins**		
VIM	Vimentin	**−122.836**
ACTA2	Actin, alpha 2, smooth muscle, aorta	**−1.639**
TNC	Tenascin C	**−18.294**
CNN2	Calponin 2	**109.554**
CALU	Calumenin	**9.943**
CAND1	Cullin associated and neddylation dissociated 1	**−50.592**
CANX	Calnexin	**289.77**
CFL1	Cofilin 1	**−4.993**
CTNNA1	Catenin alpha 1	**15.851**
CTNNA2	Catenin alpha 2	**−46.852**
CTNND1	Catenin delta 1	**−50.914**
GSN	Gelsolin	**−9.378**
KTN1	Kinectin 1	**−27.16**
VCL	Vinculin	**2.965**
THBS1	Thrombospondin 1	**−26.041**
TAGLN2	Transgelin 2	**3.578**
TAGLN3	Transgelin 3	**−18.464**
CUL1	Cullin 1	**−24.7**
CUL2	Cullin 2	**−3.6**
CUL3	Cullin 3	**−43.342**
CUL7	Cullin 7	**−24.685**
CUL4A	Cullin 4A	**−4.282**
CUL4B	Cullin 4B	**−30.196**
DES	Desmin	**−2.734**
COL11A1	Collagen type XI alpha 1 chain	**−156.421**
COL28A1	Collagen type XXVIII alpha 1 chain	**−65.077**
COL2A1	Collagen type II alpha 1 chain	**−110.781**
COL8A1	Collagen type VIII alpha 1	**−7.542**
ACTB	Actin beta	**−378.155**
ACTBL2	Actin, beta like 2	**−100.94**
ACTC1	Actin, alpha, cardiac muscle 1	**−101.765**
ACTG1	Actin gamma 1	**4.483**
ACTN1	Actinin alpha 1	**−4.133**
ACTN2	Actinin alpha 2	**−2.295**
ACT	Actin-like protein (ACT) gene	**−20.682**
ACTG1P4	Actin gamma 1 pseudogene 4	**−2.329**
ACTN4	Actinin alpha 4	**30.372**
MYH9	Myosin, heavy chain 9, non-muscle	**820.527**
MYH10	Myosin, heavy chain 10, non-muscle	**7.969**
MYH11	Myosin heavy chain 11	**−46.952**
MYH7B	Myosin heavy chain 7B	**−13.017**
MYL1	Myosin light chain 1	**−10.816**
MYL6	Myosin light chain 6	**1.94**
MYL12A	Myosin light chain 12A	**2.173**
MYL6B	Myosin light chain 6B	**–Infinity**
MYO1B	Myosin IB	**1.551**
MYO1C	Myosin IC	**−51.931**
TUBA8	Tubulin alpha 8	**−15.245**
TUBA1A	Tubulin alpha 1a	**1.545**
TUBA3E	Tubulin alpha 3e	**1.5**
TUBA4A	Tubulin alpha 4a	**−22.152**
TUBAL3	Tubulin alpha like 3	**−3.393**
TUBB3	Tubulin beta 3 class III	**−3.965**
TUBB6	Tubulin beta 6 class V	**−18.369**
TUBB8	Tubulin beta 8 class VIII	**1.78**
TUBB2A	Tubulin beta 2A class IIa	**−2.783**
TUBB2B	Tubulin beta 2B class IIb	**2.353**
TUBB4A	Tubulin beta 4A class IVa	**2.216**
TUBGCP2	Tubulin gamma complex associated protein 2	**–Infinity**
TUBGCP6	Tubulin gamma complex associated protein 6	**–Infinity**
TUBA1B	Tubulin alpha 1b	**−5.529**
TUBA1C	Tubulin alpha 1c	**−11.125**
TUBB	Tubulin beta class I	**−90.993**
TUBB4B	Tubulin beta 4B class IVb	**−25.109**
DNM1	Dynamin 1	**−20.927**
DNM2	Dynamin 2	**−18.654**
DNM3	Dynamin 3	**−9.012**
DNMBP	Dynamin binding protein	**−4.552**
LAMA3	Laminin subunit alpha 3	**−58.838**
LAMB2	Laminin subunit beta 2	**−12.831**
LAMB3	Laminin subunit beta 3	**−77.639**
LAMB4	Laminin subunit beta 4	**−8.215**

*Fold change of proteins detected in MCF7-MFF vs. MCF7-Control cells. Red: Up-regulated proteins; Green: Down-regulated proteins. Proteomics was performed as described in Materials and Methods. Statistical analyses were performed using ANOVA and 1.5-fold-changes in proteins with a p < 0.05 were considered. Dataset containing proteins with significant altered expression profile were imported into the Ingenuity Pathway Analyses (IPA) software, which groups the differentially expressed proteins into known functions and pathways*.

**Table 7:** Significant changes in other protein levels in MCF7 cells over-expressing mitochondrial fission factor (MFF).

**Symbol**	**Gene name**	**Fold change**
**Other proteins**		
MTA1	Metastasis associated 1	**−41.671**
MTA2	Metastasis associated 1 family member 2	**−17.064**
MTSS1L	Metastasis suppressor 1 like	**2.028**
UCP3	Uncoupling protein 3	**−62.533**
TIGAR	TP53 induced glycolysis regulatory phosphatase	**−39.761**
TGFBI	Transforming growth factor beta induced	**–Infinity**
TGFBR3	Transforming growth factor beta receptor 3	**−8.57**
TGIF2LX	TGFB induced factor homeobox 2 like, X-linked	**−12.818**
STAT1	Signal transducer and activator of transcription 1	**−9.228**
STAT3	Signal transducer and activator of transcription 3	**−7.953**
OXSR1	Oxidative stress responsive 1	**6.331**
PCNA	Proliferating cell nuclear antigen	**3.128**
HSF1	Heat shock transcription factor 1	**−28.884**
TRAP1	TNF receptor associated protein 1	**−10.989**
CAT	Catalase	**−10.918**
SOD2	Superoxide dismutase 2, mitochondrial	**−33.389**
TXNRD1	Thioredoxin reductase 1	**−21.824**
GLRX3	Glutaredoxin 3	**−1.845**
GSR	Glutathione-disulfide reductase	**−1E+08**
GSS	Glutathione synthetase	**2.547**
GSTM3	Glutathione S-transferase mu 3	**18.065**
ROMO1	Reactive oxygen species modulator 1	**1.966**
SMAD9	SMAD family member 9	**−1.975**
TXNL1	Thioredoxin like 1	**−9.869**
TXNRD2	Thioredoxin reductase 2	**−3.538**
TXNRD3	Thioredoxin reductase 3	**–Infinity**
TMX1	Thioredoxin related transmembrane protein 1	**3.142**
TMX4	Thioredoxin related transmembrane protein 4	**1.518**
TNFRSF12A	TNF receptor superfamily member 12A	**–Infinity**
TNFSF13B	Tumor necrosis factor superfamily member 13b	**2.397**
TRAF2	TNF receptor associated factor 2	**−7.759**

*Fold change of proteins detected in MCF7-MFF vs. MCF7-Control cells. Red: Up-regulated proteins; Green: Down-regulated proteins. Note down-regulation of relevant proteins involved in the metastatic process and oxidative stress response. Proteomics was performed as described in Materials and Methods. Statistical analyses were performed using ANOVA and 1.5-fold-changes in proteins with a p < 0.05 were considered. Dataset containing proteins with significant altered expression profile were imported into the Ingenuity Pathway Analyses (IPA) software, which groups the differentially expressed proteins into known functions and pathways*.

## Discussion

Here, we have generated a mitochondrial fission factor (MFF)-overexpressing breast cancer cell line in order to dissect the role of aberrant mitochondrial fission in the maintenance and dissemination of breast CSCs. We have found that MFF overexpression inhibits mitochondrial biogenesis and oxidative metabolism, depleting intracellular ATP levels. Furthermore, MFF-overexpressing breast cancer cells exhibit a reduction in stemness properties, as evidenced by the inhibition of 3D mammosphere formation capacity and decreases in the stem cell marker ALDH. Consistent with these observations, proteomic analysis performed in MFF-overexpressing breast cancer cells predicted a proteomic signature associated with the inhibition of mitochondrial function, and a metabolically quiescent cell phenotype, as well as the inhibition of cancer cell survival and the increase of cell death.

Mitochondria, which have classically been considered as the cell's “power-house,” extract energy from oxidative phosphorylation to efficiently support tumor growth (1). Despite their unquestionable role in generating ATP from multiple fuels, mitochondria are now considered as central hubs which drive important tumor traits. For instance, mitochondria have been implicated in metastasis formation and spreading to distant sites, as well as drug resistance (18). In addition, mitochondria have been shown to support the survival of dormant tumor cells after oncogene ablation, thus promoting disease relapse (32). Many of these actions attributed to mitochondria have been correlated with the impact that these organelles exert on the biology of CSCs (33). Several lines of evidence have suggested that mitochondria support stem cell traits, thus facilitating self-renewal and resistance to differentiation (2). For instance, a strict reliance on young, viable and competent mitochondria is required during asymmetric cell division; in fact, older and aged mitochondria are clustered within daughter cells committed to differentiation, whereas “newly synthesized” and younger mitochondria are apportioned within daughter cells that retain a stem-like phenotype (34).

As the relationship between the mitochondrial-dependent bioenergetic responses and the CSC metabolic profile has been explored, it's not surprising that a mitochondria-centric regulation of cancer energy pathways can drive complex decision-making events, ultimately impacting CSC fate toward tumor progression. As a consequence of these findings, synthetic as well as natural compounds that impair mitochondrial function have been shown to selectively hamper CSC propagation in diverse tumor types (30, 35–38). Interestingly, as mitochondria evolutionary derive from the engulfment of an α-proteobacterium in eukaryotic host cells, several classes of FDA-approved antibiotics have been suggested in a repurposing effort for the selective targeting of mitochondria (12, 39). The most remarkable of these repurposing strategies is represented by the antibiotic Doxycycline, a relatively manageable and safe tetracycline analog, which inhibits CSC dissemination *in vitro*, by targeting mitochondria biogenesis, as well as mitochondrial-dependent bioenergetic metabolism (11). Of note, a Phase II clinical trial performed in early breast cancer patients, using the oral administration of Doxycycline, was sufficient to selective reduce the stemness markers ALDH1 and CD44 (9). These studies pave the way for further exploring the role of mitochondria in cancer, and the investigation of novel mitochondrial-based druggable targets to be exploited as anti-cancer strategies.

Mitochondrial fission and fusion dynamics play an integral role in the complex regulation of mitochondrial function (25). Fragmentation and fusion are tightly balanced processes which probably derive from the same endowment mechanisms through which bacteria were co-opted into host cells. As such, it's not surprising that alterations in fusion/fission balance may play a key role in the pathogenesis of several diseases, including cancer (16, 25).

Our data show that in breast cancer enhanced mitochondrial fragmentation reduces mitochondrial mass and membrane potential, suggesting that aberrant fission may compromise the efficiency of the energetic cell machinery. Mechanistically, this model could be explained by the bi-directional link existing between mitochondrial morphology and redox homeostasis. In fact, pro-fission programs leading to mitochondrial fragmentation have been shown to stimulate ROS production (40); furthermore, the activation of mitochondrial fission was required to generate ROS during hyperglycemic conditions (41), thereby suggesting that unopposed mitochondrial fragmentation is associated with enhanced ROS production and compromission of the energetic machinery.

Indeed, analysis of metabolic flux showed that in MFF-overexpressing breast cancer cells the capability to extract ATP from energetic sources is compromised. These data are in accordance with previous studies showing that natural or synthetic compounds that block mitochondrial biogenesis and thereby reduce mitochondrial mass, also interfere with mitochondrial energetic function [reviewed in (8)]. Likewise, the metabolic effects of abnormal mitochondrial fission may parallel the biochemical responses observed in breast cancer cells treated with mitochondrial-targeting agents. Our data from proteomic analyses provide supporting evidence that MFF over-expression mainly drives the acquisition of a metabolically suppressed cell phenotype, as demonstrated by the drastic reduction of proteins involved in several energetic pathways in MCF7-MFF vs. MCF7-Control cells. Therefore, unopposed mitochondrial fragmentation induces mitochondrial dysfunction, leading to the inability to extract ATP from fuels and an overall block in cell metabolism.

As cancer cells are strictly dependent on active energetic cellular machinery for their survival, any alteration in mitochondrial integrity may compromise cancer cell viability (42, 43). The proteomic analyses performed in MFF-overexpressing breast cancer cells showed that enhanced mitochondrial fission is associated not only with loss of mitochondrial proteins, impaired ability to cope with oxidative stress, and reduced metabolic function, but also with a quiescent cell phenotype. More specifically, the proteomic landscape of MFF over-expressing breast cancer cells highlights a clear inhibition of proteins involved in cell adhesion and cell junction, together with the inhibition of the EMT program, and metastasis-related mediators. Furthermore, our proteomic analysis predicts a substantial inhibition of cell survival pathways and the activation of cell death programs, consistent with a metabolically suppressed cell phenotype.

These data are in accordance with previous studies showing that MFF-dependent mitochondrial fission activates signaling pathways involved in the inhibition of cell viability and the activation of the apoptotic response (44). In particular, MFF and other mediators involved in mitochondrial fragmentation such as DRP1 play an integral role in cancer cell death in response to diverse stimuli, including several anticancer drugs (45, 46). At least some of these effects could be attributed to the generation of mitochondrial ROS, which would thereafter trigger apoptotic cell death (47).

Mitochondrial proteins are overexpressed in CSCs, which also exhibit increased mitochondrial metabolic function (2, 5, 7). Indeed, tracking mitochondrial mass represents an important metabolic tool to identify an enrichment of CSCs (7). Due to these unusual features, CSCs are selectively targeted by a number of mitochondria impairing agents, which have been shown to compromise mitochondrial function and thereafter halt CSC propagation (8). Likewise, in the present study we have shown that mitochondrial-driven impairment in metabolic function is associated with the loss of stemness features in MFF over-expressing breast cancer cells. These observations indicate that uncontrolled mitochondrial fission may drive the acquisition of an inactive metabolic phenotype associated with the starvation of the CSCs population. Therefore, the pharmacological manipulation of mitochondrial fission may serve as an actionable tool to eradicate cancer cells, with tumor initiating capabilities. Accordingly, it has been recently shown that an MFF peptidomimetic strategy elicits anti-cancer activity in patient-derived xenografts, primary breast and lung adenocarcinoma 3D organoids, as well as glioblastoma neurospheres (45).

Recently, drugs targeting mitochondrial dynamics are beginning to emerge as novel strategies in cancer treatment. It should be mentioned that their potential is still challenged by several controversies and awaits further confirmation. For instance, the inhibitor of mitochondrial fission named mdiv-1, which acts as a Drp1 GTPase activity inhibitor, can cause a loss of the functional properties of CSCs (48). However, this drug appears to also work as a reversible inhibitor of mitochondrial complex I, leading to alterations of mitochondrial ROS production (49). Additional layers of complexity have to be considered when exploring the contribution of mitochondrial shaping genes in tumor biology. Indeed, tumor microevironmental conditions; the presence of hormonal/growth factors; and intrinsic features of the specific tumor cell types involved may affect mitodynamics pathways.

In conclusion, the data presented herein validate the use of a mitodynamic approach to target mitochondria architecture and function toward the eradication of CSCs. Further studies will be necessary to validate the use of this therapeutic strategy, especially in combination with additional metabolic inhibitors as novel tools to control stem traits in cancer.

## Data Availability Statement

The datasets generated for this study can be found in online repositories. The names of the repository/repositories and accession number(s) can be found here: ProteomeXchange via PRIDE dataset (identifier: PXD020802).

## Author Contributions

ML and FS conceived and initiated this project. All experiments described in this paper were performed by RS-A. MF performed the 3D mammosphere assays. ED wrote the manuscript with input from RS-A. ML and FS edited the manuscript. All authors contributed to the article and approved the submitted version.

## Conflict of Interest

ML and FS hold a minority interest in Lunella Biotech, Inc. The remaining authors declare that the research was conducted in the absence of any commercial or financial relationships that could be construed as a potential conflict of interest.
